# Inner Ear Mixed Hearing Loss

**DOI:** 10.1055/s-0044-1786830

**Published:** 2024-07-05

**Authors:** Pedro Luiz Mangabeira Albernaz

**Affiliations:** 1 Department of Surgery, Hospital Israelita Albert Einstein, São Paulo, SP, Brazil

In 1971, Farrior and Endicott 1 mentioned that there were 22 reported cases of congenital hearing loss associated with enlargement of the cochlear aqueduct. They added six new cases. Some of these patients were submitted to stapedectomies, in an attempt to eliminate the conductive part of the hearing loss. They had gushers, with a large flow of cerebrospinal fluid filling the operating field when the stapes footplate was punctured or removed. The hearing became better for a short time, but this improvement did not last. They attributed this hearing loss to an enlarged cochlear aqueduct or to a defect in the modiolus. Recurrent otic meningitis due to footplate fistulas have also been reported by Stool et al., 2 Hipskind et al, 3 Gacek and Leipzik, 4 and Jensen et al. 5

In 1973, Glasscock 6 published an extensive review of this problem, and reported 3 cases that he had operated using the technique described by Farrior for the ablation of the cochlear aqueduct. The operations did not change the hearing thresholds of these patients.

In 1992, I 7 published a study of 9 patients presenting what I called **perilymphatic hypertension** . None of them presented otic meningitis. It was interesting to note that these patients, in spite of the mixed hearing loss, presented tympanic reflexes; the perilymphatic hypertension increases the rigidity of the ossicular chain. This is a very important finding for the diagnoses of these patients and to minimize the chances of getting cerebrospinal fluid gushers. This paper 7 was the first to describe the presence of tympanic reflexes in mixed hearing losses.

In 1998, Minor et al. 8 described a different cause for mixed hearing loss: due to superior canal dehiscence. This defect is probably congenital, but the first symptoms appear around 40 years of age.

The intensity of the symptoms is quite variable; the most common are autophony, tinnitus, oscillopsia, discomfort in the presence of intense sounds, and occasional changes in intracranial pressure. Since there is a fistula in the perilymphatic space, one can conclude that this syndrome is linked to **perilymphatic hypotension** .

Imaging is quite important, as it can show enlarged cochlear aqueducts or superior canal dehiscence. But the tomographic scans may be normal in the cases with modiolus defects. Curiously, both perilymphatic hypertension and hypotension are linked to mixed hearing loss with the presence of tympanic reflexes.

In conclusion, mixed hearing losses with the presence of tympanic reflexes are always related to inner ear problems.
